# Dementia in the Middle East and North Africa: an integrative narrative review of epidemiology, risk structure, and health system implications

**DOI:** 10.7189/jogh.16.04181

**Published:** 2026-07-17

**Authors:** Iman Dajani, Anns Mahboob, Shahad Sabaawi Ibrahim, Raghad Sabawi Ibrahim, Mohsen Sedighi, Zahra Shirzadi, Simin Mahinrad, Serena Sabatini, Ahmad R Khatoonabadi, Matthew Prina, Carolina B Castro, Jessica L Mantegna, P Anthony Akkari, Akram A Hosseini, Ralph N Martins, Claire V Burley, Jennifer Dunne, Blossom CM Stephan, Hamid R Sohrab, Ali Chaari

**Affiliations:** 1 Weill Cornell Medicine Qatar, Qatar Foundation, Education City, Doha, Qatar; 2 Trauma and Injury Research Center, Iran University of Medical Sciences, Tehran, Iran; 3 Department of Neurology, Massachusetts General Hospital, Harvard Medical School, Cambridge, Massachusetts, USA; 4 Medical & Scientific Relations, Alzheimer's Association, Chicago, Illinois, USA; 5 Department of Clinical Psychology and Psychobiology, University of Barcelona, Barcelona, Spain; 6 School of Psychology, University of Surrey, Guildford, Surrey, UK; 7 School of Rehabilitation, Tehran University of Medical Sciences, Tehran, Iran; 8 Population Health Sciences Institute, Faculty of Medical Sciences, Newcastle University, Newcastle Upon Tyne, UK; 9 School of Psychology, Murdoch University, Murdoch, Perth, Western Australia, Australia; 10 Centre for Healthy Ageing, Health Futures Institute, Murdoch University, Murdoch Murdoch, Perth, Western Australia,Australia; 11 Perron Institute for Neurological and Translational Science, Ralph & Patricia Sarich Neuroscience Institute, Perth, Western Australia,, Australia; 12 School of Health Sciences, Institute for Health Research, The University of Notre Dame Australia, Fremantle Western Australia, Australia; 13 Personalised Medicine Centre, Health Futures Institute, Murdoch University, Murdoch, Perth, Western Australia, Australia; 14 School of Human Sciences, University of Western Australia, Crawley, Perth, Western Australia, Australia; 15 Department of Academic Neurology, Queen’s Medical Centre, Nottingham University Hospitals NHS Trust, Nottingham, UK; 16 Sir Peter Mansfield Imaging Centre, University of Nottingham, Nottingham, UK; 17 Faculty of Medicine, Health and Human Sciences, Macquarie University, Sydney, New South Wales, Australia; 18 Dementia Centre of Excellence, enAble Institute, Curtin University, Perth, Western Australia, Australia

**Keywords:** dementia, Alzheimer’s disease, Middle East and North Africa, modifiable risk factors, socioeconomic disparities, prevention strategies

## Abstract

**Background:**

Dementia prevalence in the Middle East and North Africa (MENA) is projected to rise exponentially, with estimates suggesting increases of 367% by 2050. This region faces distinctive challenges, including rapid demographic transition, high rates of modifiable risk factors, and fragmented health systems. This paper examines region-specific risk factors and pooled prevalence rates of dementia across the MENA region.

**Methods:**

We conducted a narrative review of dementia risk factors in the MENA region, including analysis of population-attributable risk (PAR) estimates and pooled estimates of dementia prevalence among adults aged 50 years and older.

**Results:**

Dementia prevalence was estimated at 1.84% among individuals aged 50 years and older, increasing to 3.81% among those aged 60 years and older, with substantial variability across countries. Smoking (>30%), physical inactivity ( ~ 50%), and high burdens of diabetes, hypertension, and obesity were consistently associated with increased dementia risk. Additional region-specific drivers include nutritional transitions, gut dysbiosis, consanguinity, and environmental risk factors such as air pollution and climate-related heat stress. Critical barriers to prevention and care include limited research infrastructure, low health literacy, stigma, and inequitable health care access.

**Conclusions:**

Dementia prevalence in MENA is rising rapidly, driven by demographic change and high exposure to modifiable risk factors that are relatively region-specific in terms of their contribution. Strengthening prevention, early detection, and equitable access to care should be regional health priorities.

Dementia ranks among the century’s most pressing public-health threats currently affecting over 55 million people globally and expected to rise to about 152 million by 2050 [1]. The global cost was estimated at 1.31 trillion USD in 2019, nearly half from informal caregiving, underscoring the immense social and economic impact of the disease [2].

The Middle East and North Africa (MENA) region is poised to experience one of the steepest increases in dementia worldwide, with model-based projections estimating that, under current demographic trajectories, dementia prevalence in parts of the MENA region could increase by approximately 367% by 2050, largely driven by population aging and demographic expansion [3]. In 2021, approximately 1.33 million people in MENA countries were living with dementia, generating annual costs of 10.4 to 13.9 billion USD [4]. Unlike high-income countries where formal medical and social care dominate expenditures, the MENA region relies heavily on informal caregiving, reflecting cultural norms, limited institutional capacity, and gender inequities [5].

The escalating dementia burden in the MENA region is exacerbated by several region-specific determinants. Notably, the region exhibits some of the world’s highest prevalences of diabetes, obesity, and tobacco use. In conjunction with rapid nutritional transitions and comparatively low educational attainment, these factors markedly increase susceptibility to dementia [6,7]. Additional challenges include high consanguinity, environmental exposures such as chronic air pollution and extreme heat, and pervasive stigma that delays diagnosis and care[3,8,9]. However, research infrastructure remains limited, with few longitudinal cohorts or biomarker studies and little integration of dementia into national non-communicable disease (NCD) strategies [3].

This integrative narrative review aims to:

(1) synthesise current evidence on dementia prevalence and risk factors across the MENA region,

(2) examine how region-specific demographic, cardiometabolic, environmental, and sociocultural factors interact to shape dementia trajectories, and

(3) contextualise these findings within health system structures and economic patterns to inform prevention and policy prioritisation.

While drawing on global frameworks such as the 2024 Lancet Commission on Dementia [1], this review applies a regional analytic lens to identify how the convergence of cardiometabolic burden, sociocultural caregiving structures, environmental exposures, and uneven health system capacity creates a distinct dementia risk architecture in MENA compared with high-income settings.

## METHODS

### Search strategy

We searched PubMed, Scopus, and Google Scholar for studies published between 1 January 2000 and 31 December 2024. Search queries used Boolean combinations of keywords, including (‘dementia’ OR ‘Alzheimer disease’ OR ‘vascular dementia’) AND (‘Middle East’ OR ‘North Africa’ OR individual country names) AND (‘prevalence’ OR ‘incidence’ OR ‘risk factors’ OR ‘epidemiology’). Searches were adapted to the syntax requirements of each database. The search yielded a substantial number of records across databases. After screening titles and abstracts for relevance, full-text articles were reviewed based on predefined inclusion criteria. Given the integrative narrative design, the search process was iterative and supplemented by manual reference screening. We did not apply formal risk-of-bias scoring tools, as the objective was broad synthesis rather than pooled inferential estimation.

### Eligibility criteria and study selection

To situate the MENA findings in a global context, we reviewed the international literature. For cost comparisons, we used the most recent worldwide estimates from Wimo et al. (2019 data; published 2023) [2] and regional estimates from Qassem et al. (2021 data; published 2023) [4], which focused on Arab countries within MENA. Cost estimates from Qassem et al. apply specifically to Arab MENA countries and therefore exclude non-Arab countries within the broader World Bank MENA classification (*e.g*. Israel, Turkey, and Iran). Where applicable, this distinction is noted to ensure denominator consistency. To ensure comparability, costs were classified into direct (medical and social) and indirect (informal caregiving) categories. In areas with limited regional data, we referenced the World Alzheimer Reports and the 2024 Lancet Commission on Dementia[1,10]. Only English-language publications were included. We acknowledge that this may introduce language bias in a multilingual region and may underrepresent locally published data.

### Evidence synthesis approach

We did not perform formal risk-of-bias scoring or quantitative heterogeneity metrics (*e.g*. *I*^2^) because the pooled analyses were conducted for descriptive regional approximation rather than inferential meta-analytic purposes. Given substantial variability across studies in diagnostic criteria (*e.g*. DSM *vs*. ICD *vs*. screening instruments), sampling frames (community-based *vs*. hospital-based), and age stratification across countries, findings were synthesised narratively. Such heterogeneity in case definitions and ascertainment methods has been widely documented in global dementia epidemiology literature [11]. Pooled prevalence estimates were generated using random-effects models for age thresholds ≥50 and ≥60 years where sufficient comparable data were available. Because of heterogeneity in case definitions, diagnostic pathways, and underdiagnosis across settings, these pooled estimates should be interpreted as approximate regional summaries rather than precise population-level measures.

Population-attributable risk (PAR) estimates were calculated using standard Levin’s formula, incorporating exposure prevalence from regional epidemiologic data and relative risk estimates derived from established global meta-analyses and longitudinal cohort studies (*e.g*. Lancet Commission and related syntheses). Because many cardiometabolic risk factors (*e.g*. obesity, diabetes, hypertension) are intercorrelated, PAR estimates were calculated and presented individually and were not summed to derive a joint regional PAR. Accordingly, figures illustrate potential relative contributions rather than cumulative attributable burden and should be interpreted as indicative rather than additive.

Although random-effects models were used to generate pooled prevalence estimates, this review was not designed as a formal systematic review or meta-analysis. Given heterogeneity in study design, case definitions, and sampling strategies across countries, pooled estimates are presented as exploratory quantitative summaries intended to approximate regional central tendencies rather than to provide definitive population-level estimates. Accordingly, findings should be interpreted cautiously and within the broader narrative synthesis framework.

### Epidemiology

The World Health Organization (WHO) estimated that more than 55 million people worldwide were living with dementia in 2020 with an annual global cost of approximately 1.3 trillion USD [12]. By 2050, this number is projected to triple to 153 million, with nearly 71% of cases expected to occur in low- and middle-income countries (LMICs), up from 60% in 2020 [13]. Contributing factors to this trend include increasing life expectancy, higher exposure to risk factors (including poverty), and limited health care access [13]. In 2021, approximately 1.33 million individuals were living with dementia across MENA, with estimated care costs ranging from 10.4 to 13.9 billion USD [4].

Geographically, the MENA region captures 23 countries ranging from LMICs (*i.e*. Afghanistan, Algeria, Egypt, Iran, Iraq, Jordan, Lebanon, Libya, Morocco, Mauritania, Oman, Sudan, Syrian Arab Republic, Tunisia, West Bank and Gaza, and Yemen) to high-income countries (*i.e*. Bahrain, Israel, Kuwait, Qatar, Saudi Arabia, Turkey, United Arab Emirates) [14]. Despite relatively shared elements in culture, climate, and lifestyle, the region exhibits significant economic and health system variability. Dementia prevalence varies widely – from 2% in Qatar to 4% in Tunisia among individuals aged ≥60 (**Table 1**) [4,18,19]. However, regional trends show a consistent increase in dementia burden (Figure S1 in the **Online Supplementary Document**). Model-based demographic projections suggest that relative to 2019 baseline estimates, dementia prevalence could increase by approximately 240–280% in some countries and up to 340–2000% in smaller or rapidly aging populations by 2050. These projections reflect population aging, demographic expansion, and current risk factor distributions rather than deterministic forecasts, and are contingent on underlying modelling assumptions [18,19].

**Table 1:** The prevalence of dementia across the MENA countries with corresponding evidence

Country*	Prevalence (%)	Year	Reference
Algeria	Age >50: 2.23	2023	[15]
	Age >60: 4.19		
Bahrain	Age >50: 1.49	2023	[15]
	Age >60: 3.47		
Egypt	Age >50: 1.96	2023	[15]
	Age >60: 3.78		
Iran	Age 60–64: 3.7	2016	[16]
	Age 65–69: 6.2		
	Age 70–74: 10.4		
	Age 75–79: 14.4		
	Age >80: 13.0		
Iraq	Age >50: 1.86	2023	[15]
	Age >60: 3.93		
Israel	Age >45: 2.5	2016	[17]
	Age >65: 6.4		
Jordan	Age >50: 1.80	2023	[15]
	Age >60: 3.89		
Kuwait	Age >50: 1.19	2023	[15]
	Age >60: 2.99		
Lebanon	Age >50: 2.83	2023	[15]
	Age >60: 4.88		
Morocco	Age >50: 2.16	2023	[15]
	Age >60: 3.84		
Oman	Age >50: 1.74	2023	[15]
	Age >60: 3.84		
Qatar	Age >50: 0.91	2023	[15]
	Age >60: 2.84		
Saudi Arabia	Age >50: 1.32	2023	[15]
	Age >60: 3.65		
Syria	Age >50: 2.09	2023	[15]
	Age >60: 4.05		
Sudan	Age >50: 1.93	2023	[15]
	Age >60: 3.47		
Tunisia	Age >50: 2.50	2023	[15]
	Age >60: 4.09		
United Arab Emirates	Age >50: 1.33	2023	[15]
	Age >60: 4.09		
Yemen	Age >50: 2.02	2023	[15]
	Age >60: 3.98		

MENA – Middle East and North Africa

*Libya and West Bank and Gaza are not included due to the lack of recently published data.

At the same time, true prevalence is almost certainly underestimated. Most available data are derived from hospital-based samples, small-scale community surveys, or extrapolations from global models. Few population-based cohort studies exist, and most countries in the region do not have a national dementia registry. Underdiagnosis is compounded by stigma, low dementia literacy among families and health care providers, and limited access to paraclinical diagnostic services including neuroimaging, Alzheimer disease (AD) fluid biomarkers laboratories and neuropsychological testing [5,10]. These gaps are particularly pronounced in rural areas, where health system infrastructure is far weaker and illiteracy rates remain very high. Epidemiological inequities also shape dementia patterns in MENA. Lower educational attainment, especially among older women, reduces cognitive reserve and the likelihood of following healthier lifestyles and may partly explain differences in prevalence across countries [6]. Furthermore, socioeconomic disparities limit access to timely diagnosis and formal care, reinforcing dependence on informal caregiving networks. Together, these factors suggest that current estimates represent a conservative picture of the actual dementia burden in MENA.

**Figure 1:**
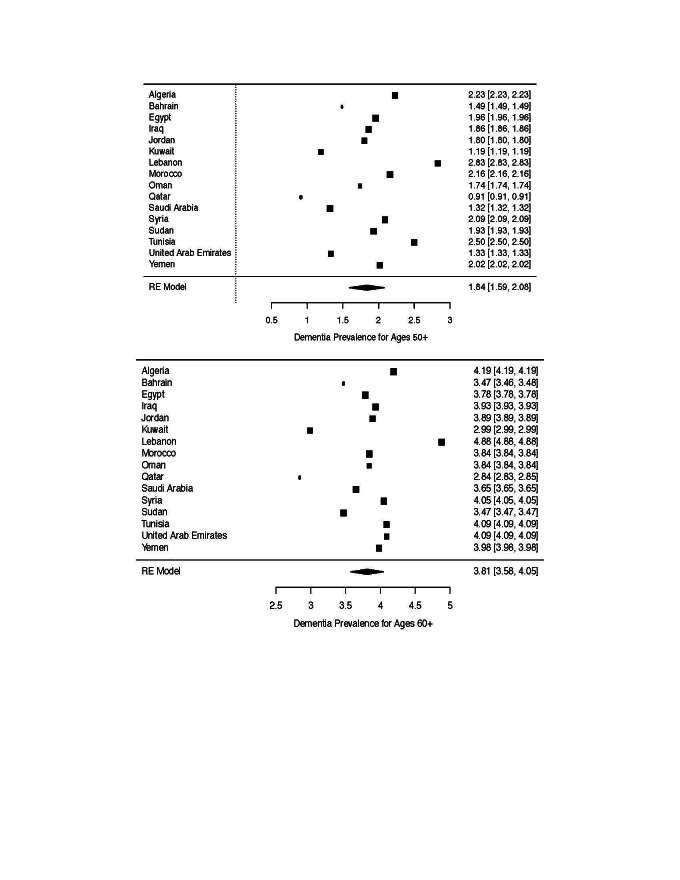
Panel A. Forest plot showcasing dementia prevalence in individuals aged ≥50 across MENA countries using random effect (RE) model. Panel B. Forest plot showing the prevalence of dementia in individuals aged ≥60 across the MENA countries using RE model. Pooled estimates were generated using random-effects models; heterogeneity across studies in diagnostic criteria and sampling frames should be considered when interpreting these estimates. MENA – Middle East and North Africa

Published prevalence estimates from MENA were synthesised, and where possible, generated pooled estimates using random-effects models of the prevalence rates of dementia in the region (excluding Iran and Israel from pooled estimation due to lack of directly comparable age-stratified community-based prevalence data consistent with the ≥50 and ≥60 age thresholds used in the pooled models) using the restricted maximum likelihood estimation and the Knapp-Hartung adjustment for hypothesis testing. Unless otherwise specified, prevalence estimates refer to adults aged ≥50 or ≥60 years as defined in the pooled analyses. Exploratory pooled analysis suggested a regional prevalence of approximately 1.84% (95% CI = 1.57–2.10) in individuals aged ≥50 (**Figure 1**, Panel A), increasing to 3.81% (95% CI = 3.56–4.07) in those aged ≥60 (**Figure 1**, Panel B).

These pooled estimates reflect aggregated signals across heterogeneous studies and should not be interpreted as implying uniform prevalence across countries. Wide differences in study methodology, diagnostic pathways, and underdiagnosis across settings limit direct comparability. The confidence intervals reflect statistical dispersion within the model but do not fully capture structural heterogeneity across health systems and study designs in the region. Additionally, exclusions were based on methodological comparability rather than epidemiologic considerations. Available data from excluded countries differed in age categorisation, sampling frame, or reporting format in ways that precluded inclusion within the exploratory pooled model without introducing additional structural heterogeneity. Their exclusion does not imply lower or higher burden but reflects constraints in cross-study comparability.

### Modifiable risk factors of dementia

Dementia is associated with both non-modifiable and modifiable risk factors, of which increasing age is the predominant risk factor [13]. The 2024 Lancet Commission on Dementia identified 14 modifiable risk factors across the life course: low educational attainment in early life; hearing loss, hypertension, obesity, smoking, depression, physical inactivity, diabetes, excessive alcohol consumption, traumatic brain injury, and high low-density lipoprotein (LDL) cholesterol during midlife; and social isolation, visual impairment, and air pollution in later life. Collectively, these factors account for ~ 45% of dementia risk globally [1]. Of these, 11 are especially pertinent to MENA due to high prevalence: smoking, low levels of education, hypertension, obesity, diabetes, physical inactivity, hearing loss, high cholesterol, air pollution, and infrequent social contact. In addition, we examined dietary patterns, gut microbiota health, and consanguinity, given their emerging links to dementia risk in this region.

### Lifestyle and behavioural risk factors

#### Smoking

Smoking prevalence in MENA is among the highest globally, with rates exceeding 30% in many countries, compared to 10–12% in high-income settings [7]. Notably, rates are markedly higher in men ( ~ 50%) than women ( ~ 5%). The lowest prevalence is observed in Maghreb (Algeria, Morocco, Libya, Tunisia, and Mauritania) countries, while Lebanon and other Eastern Mediterranean countries report the highest [7]. Gender and cultural patterns influence tobacco use in the region. Women generally report low rates due to social norms, but waterpipe (shisha) smoking has grown in popularity, especially among younger populations and women, and is often wrongly perceived as less harmful despite delivering similar or greater toxic exposures [20].

Smoking is associated with increased risk of both AD and vascular dementia (VaD), with heavy smoking (>20 cigarettes/d) conferring a 34% higher risk [21]. Proposed mechanisms include promotion of atherosclerosis, impaired cerebral blood flow, oxidative stress, and neuroinflammation, which have been implicated in pathways linked to neurodegeneration [21]. Former smokers show lower risk of dementia compared to current smokers, underscoring the benefits of cessation. Despite global declines of 27–38% in tobacco use since 1990 [22], progress in MENA has been limited. Several countries lack comprehensive tobacco control policies or face weak enforcement, limiting the effectiveness of public health interventions.

#### Physical inactivity

Physical inactivity is a major contributor to dementia risk, with protective effects observed when individuals meet international guidelines of at least 150 minutes of moderate or 75 minutes of vigorous physical activity per week. Regular physical activity promotes neurogenesis, increases gray matter volume, and strengthens neural connectivity in regions related to memory and executive function [23]. Long-term cohort studies support the role of regular physical activity in reducing the incidence of all-cause dementia and AD, even across follow-ups ≥20 years [24].

Across the MENA region, 40–50% of adults fail to achieve targets for physical activity, nearly double the global average of 27.5% [25]. The prevalence of physical inactivity is especially high in Gulf countries (*i.e*. Bahrain, Kuwait, Oman, Qatar, Saudi Arabia, and the United Arab Emirates), where sedentary lifestyles are compounded by car-oriented infrastructure, limited green spaces, and climate-related barriers such as extreme heat [26]. Women in Gulf countries are disproportionately affected by physical inactivity, with cultural norms and safety-related concerns restricting opportunities for exercise; national estimates indicate that only about 26–28% of adult women in the GCC meet recommended activity guidelines [25,27]. Despite recognition of the problem, few MENA countries have implemented large-scale, culturally tailored programmes to promote activity, underscoring a significant prevention gap.

#### Nutrition and gut microbiota

**Figure 2:**
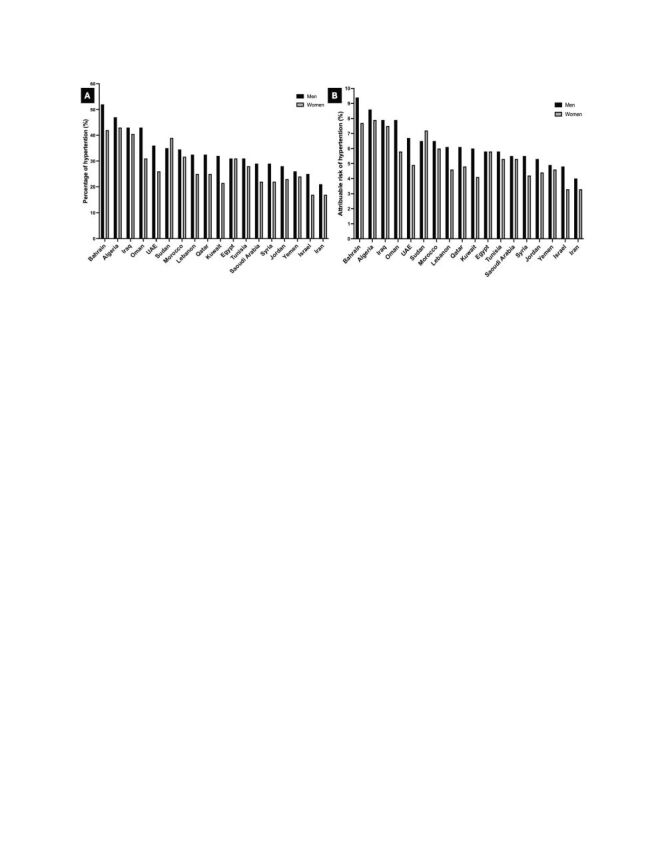
Age-standardised prevalence of hypertension (Panel A) and population attributable risk of hypertension (Panel B) for dementia in men and women aged 30–79 years (2019) in countries of MENA region. Population attributable risk (PAR) estimates were calculated using exposure prevalence and relative risk estimates derived from published global meta-analyses. PAR values assume independence of exposures and do not account for clustering effects. MENA – Middle East and North Africa, PAR – population attributable risk.

The MENA region has undergone a profound nutritional transition in recent decades, shifting away from traditional Mediterranean-style diets – historically rich in whole grains, legumes, fruits, vegetables, fish, and olive oil – toward more Western-style diet characterised by ultra-processed foods, refined sugars, and saturated fats [28,29]. Urbanisation, globalisation, and economic change have accelerated this dietary shift, contributing to rising rates of obesity, type 2 diabetes mellitus (T2DM), and hypertension, all of which are established dementia risk factors [15]. This pattern is reflected in the high prevalence of hypertension across the region, with age-standardised rates exceeding 50% in countries like Bahrain and Algeria and contributing substantially to the population-attributable risk of dementia (**Figure 2**). Beyond vascular and metabolic mechanisms, dietary quality also shapes brain health through the gut microbiota. Traditional diets rich in antioxidants, polyphenols, and omega-3 fatty acids support microbial diversity and confer neuroprotective benefits, while Western dietary patterns have been associated with gut dysbiosis and systemic inflammatory profiles, which are hypothesised to influence neuroinflammatory pathways via the microbiota-gut-brain axis [16].

It is important to note that direct longitudinal evidence from MENA populations linking microbiota alterations to incident dementia remains limited, and current understanding is largely extrapolated from mechanistic and non-regional cohort studies. Although regional microbiome studies remain limited and causal pathways remain under active investigation, available evidence suggests that adherence to traditional dietary patterns maintains healthier microbial profiles and may buffer against cognitive decline, whereas the increasing reliance on processed foods, especially among younger urban populations, may be associated with increased vulnerability to cognitive decline over time.

#### Social isolation

Social interaction is a key cognitive stimulus that enhances memory, language, and executive function by activating neural networks associated with learning and problem-solving. In contrast, social isolation has been consistently linked to increased dementia risk and accelerated cognitive decline [17]. Across the MENA region, between 20–30% of older adults (often defined as ≥65 years in global cohorts) are estimated to experience some level of social isolation [19]. Although many societies in the region have traditionally benefited from strong family networks and communal traditions that foster intergenerational interaction, these protective dynamics are being eroded by rapid urbanisation, labour migration, and shifting household structures [30].

In the Gulf states, reliance on migrant caregivers may reduce opportunities for older adults to engage directly with younger family members, weakening intergenerational bonds [31]. In conflict-affected settings such as Syria and Yemen, widespread displacement has disrupted traditional community structures, leaving many older adults socially disconnected and vulnerable [32]. Beyond structural changes, increasing reliance on digital communication over face-to-face interaction also contributes to reduced cognitive and social stimulation among older adults. These shifts underscore the importance of promoting social engagement through community programmes, intergenerational contact, and culturally appropriate interventions in aging populations.

#### Hearing loss

Research has shown a potential link between untreated, age-related hearing loss, presbycusis, and an increased risk of cognitive impairment and dementia [1]. It has been suggested that the cognitive load caused by untreated hearing loss may partially contribute to an increased risk of dementia [1]. Although there is limited research on the link between hearing loss and dementia in the MENA region compared to global studies, the region has a rapidly growing aging population with the potential risk of hearing loss. Population-based studies in the MENA region suggest that between 5–6% of adults experience some degree of hearing loss, with a Jordanian study reporting prevalence as high as 6.3% [33]. However, these figures likely underestimate the true burden, as screening programmes are limited, and milder deficits often go undetected.

Globally, hearing aid uptake remains strikingly low and only about 17% of the 400 million people with clinically significant hearing impairment have access [34]. In MENA, this gap is compounded by barriers such as cost, stigma, and limited service provision, particularly in rural and underserved settings [35]. Given the region’s rapidly aging populations, untreated hearing loss in older adults represents an under-recognised but important modifiable factor to dementia risk, with implications for both quality of life and long-term cognitive health.

#### Low levels of education

Lower education attainment is among the strongest modifiable risk factors for dementia, primarily because it contributes to reduced cognitive reserve and limited lifelong cognitive stimulation [36]. Adult literacy rates across the MENA region demonstrate striking variation: near-universal literacy is reported in countries such as Bahrain (men = 99.9%, women = 94.9%), whereas literacy remains substantially lower in countries like Sudan (men = 65.4%, women = 56.1%) (**Table 2**). Across nearly all MENA countries, female literacy lags male literacy – for example, in Yemen the gap is over 30 percentage points (men = 85.1%, women = 55.0%) (**Table 2**). Such gender disparities may partially account for the higher prevalence of dementia among women in this region. Educational inequalities in the MENA region are due to historical political instability, underinvestment in formal and vocational training, cultural barriers, and urban-rural divides [38]. A cross-sectional cohort study in Lebanon showed that higher education, complex occupational attainment, and leisure activities significantly predicted better global cognitive function and reduced dementia burden [6]. Promoting advanced education and vocational training within the MENA region, especially for women and rural populations, could enhance cognitive reserve and serve as a population-level intervention to lower dementia risk.

**Table 2:** The rates of literacy in adults aged ≥15 y across MENA countries (estimates are the latest available, extracted from World Population Literacy Review) [37]

Country	Adult literacy rate (%)	Year	Reference
Algeria	Men: 87.4	2018	[37]
	Women: 75.3		
Bahrain	Men: 99.9	2022	[37]
	Women: 94.9		
Egypt	Men: 78.8	2022	[37]
	Women: 67.4		
Iran	Men: 92.4	2022	[37]
	Women: 88.7		
Iraq	Men: 91.2	2017	[37]
	Women: 79.9		
Israel	Men: 98.7	2011	[37]
	Women: 96.8		
Jordan	Men: 98.7	2021	[37]
	Women: 98.4		
Kuwait	Men: 97.1	2020	[37]
	Women: 95.4		
Lebanon	Men: 96.9	2019	[37]
	Women: 93.3		
Morocco	Men: 84.8	2022	[37]
	Women: 67.4		
Oman	Men: 97.0	2022	[37]
	Women: 92.7		
Qatar	Men: 92.4	2017	[37]
	Women: 94.7		
Saudi Arabia	Men: 98.6	2020	[37]
	Women: 96.0		
Sudan	Men: 65.4	2018	[37]
	Women: 56.1		
Syria	Men: 91.7	2019	[37]
	Women: 81.0		
Tunisia	Men: 89.1	2021	[37]
	Women: 75.6		
United Arab Emirates	Men: 98.8	2021	[37]
	Women: 97.2		
Yemen	Men: 85.1	2021	[37]
	Women: 55.0		

MENA – Middle East and North Africa

### Cardiometabolic and vascular risk factors

#### Hypertension

Chronic hypertension is consistently associated with increased dementia risk and is believed to contribute to small-vessel disease, cerebral hypoperfusion, and cumulative vascular injury that may accelerate cognitive decline [39]. In the MENA region, prevalence ranges from 25–40% of adults, with the highest rates reported in Gulf and Levant countries (*i.e*. Syria, Lebanon, Israel, Palestine, and Jordan) (**Figure 2**). The primary hypertension risk factors in the region include sedentary lifestyles, poor dietary habits, and population aging, with many cases remaining undiagnosed until significant complications arise.

This reflects a broader regional challenge in the management of non-communicable diseases, where conditions such as hypertension are frequently underdiagnosed and poorly managed. According to WHO, awareness, treatment, and control of hypertension remain poor worldwide as fewer than half of adults with hypertension are aware of their condition, only 42% receive treatment, and just about 1 in 5 achieve blood pressure control [40]. In the MENA region, a 2021 systematic review of 178 population-based studies reported similar gaps, with approximately 50% awareness, 41% treatment, and only 19% control rates [41]. Given the strong association between midlife hypertension and late onset dementia [42], inadequate detection and management represent a critical pathway by which the dementia burden will rise across the region.

#### Diabetes

**Figure 3:**
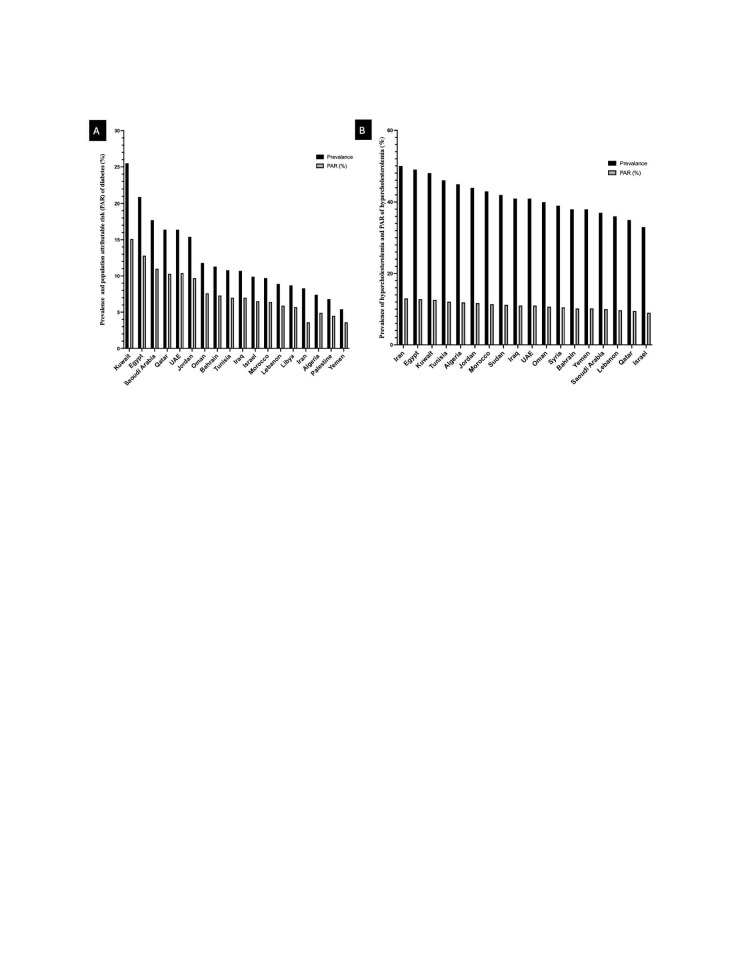
Panel A. Prevalence of diabetes and population attributable risk (PAR) of diabetes for dementia in 2021 across the MENA region. Panel B. Prevalence of hypercholesterolemia and population attributable risk of hypercholesterolemia for dementia in 2019 across MENA countries. Population attributable risk (PAR) estimates were calculated using exposure prevalence and relative risk estimates derived from published global meta-analyses. PAR values assume independence of exposures and do not account for clustering effects. MENA – Middle East and North Africa, PAR – population attributable risk.

T2DM has been associated with a 1.5–2.5 fold higher risk of dementia, particularly among adults aged ≥65 years [43]. In MENA countries, T2DM often develops at a younger age than in other regions (**Figure 3**, Panel A), resulting in a longer lifetime exposure to metabolic and vascular injury that compounds dementia risk [44]. The region now has some of the highest diabetes prevalence worldwide, with Gulf countries reporting rates exceeding 20% of adults [37], and projections suggesting further increases alongside rising obesity rates.

Diabetes has been hypothesised to influence dementia risk through several interrelated mechanisms, including vascular damage, chronic inflammation, and insulin resistance, which impairs neuronal glucose uptake and accelerates neurodegeneration. Its atherogenic effects further increase the likelihood of cerebral plaque formation, ischemic events, and VaD [45]. The earlier onset and rapid escalation of T2DM in MENA thus not only elevate dementia risk but also extend the period during which the brain is exposed to metabolic stress, magnifying long-term cognitive consequences

#### Obesity

Obesity is a major public health concern in the MENA region, particularly in the Gulf States and among women, where prevalence rates are among the highest, globally (**Table 3**). Global projections indicate that nearly half of all adults (around three billion people) could be overweight or obese by 2030 [46]. Given current trends of obesity prevalence in MENA countries – already among the highest globally – could similarly exceed 50% in several Gulf states within the next decade [47]. This surge can partially be explained by rapid urbanisation, nutrition transitions, and declining physical activity levels.

**Table 3:** The Prevalence of obesity and PAR of obesity for dementia across MENA countries

Country	Adult literacy rate (%)	Year	Reference
Algeria	Men: 87.4	2018	[37]
	Women: 75.3		
Bahrain	Men: 99.9	2022	[37]
	Women: 94.9		
Egypt	Men: 78.8	2022	[37]
	Women: 67.4		
Iran	Men: 92.4	2022	[37]
	Women: 88.7		
Iraq	Men: 91.2	2017	[37]
	Women: 79.9		
Israel	Men: 98.7	2011	[37]
	Women: 96.8		
Jordan	Men: 98.7	2021	[37]
	Women: 98.4		
Kuwait	Men: 97.1	2020	[37]
	Women: 95.4		
Lebanon	Men: 96.9	2019	[37]
	Women: 93.3		
Morocco	Men: 84.8	2022	[37]
	Women: 67.4		
Oman	Men: 97.0	2022	[37]
	Women: 92.7		
Qatar	Men: 92.4	2017	[37]
	Women: 94.7		
Saudi Arabia	Men: 98.6	2020	[37]
	Women: 96.0		
Sudan	Men: 65.4	2018	[37]
	Women: 56.1		
Syria	Men: 91.7	2019	[37]
	Women: 81.0		
Tunisia	Men: 89.1	2021	[37]
	Women: 75.6		
United Arab Emirates	Men: 98.8	2021	[37]
	Women: 97.2		
Yemen	Men: 85.1	2021	[37]
	Women: 55.0		

MENA – Middle East and North Africa, PAR – population attributable risk

**Correspondence to:** Ali Chaari Weill Cornell Medicine-Qatar, Qatar Foundation Education City, Al Luqta Street, Al Rayyan, Doha Qatar alc2033@qatar-med.cornell.edu Hamid Sohrabi Centre for Healthy Ageing, Health Futures Institute, Murdoch University 90 South Street, Murdoch, Western Australia Australia Hamid.Sohrabi@murdoch.edu.au

Beyond its role in increasing the risk of diabetes and hypertension, obesity has been associated with cognitive decline and may influence dementia risk through mechanisms such as systemic low-grade inflammation, insulin resistance, and vascular dysfunction, all of which accelerate neurodegenerative processes [48]. Women in MENA face additional barriers related to sociocultural expectations, restricted opportunities for physical activity, and higher baseline prevalence, amplifying the long-term risk at a population level [25]. This trend highlights the pressing need to address obesity in the region as a critical driver of dementia risk, both directly and through its amplification of cardiometabolic comorbidities.

#### Hypercholesteremia

The 2024 Lancet Commission on Dementia, for the first time, identified elevated LDL cholesterol as a modifiable risk factor, supported by emerging longitudinal evidence [1]. Large-scale cohort studies suggest that high LDL significantly increases the risk of all-cause dementia and AD, with the association being particularly strong among individuals under the age of 65 [49].

Mechanistically, elevated LDL cholesterol has been associated with increased deposition of amyloid-beta (Aβ) and tau proteins, endothelial dysfunction, and reduced cerebral perfusion in observational studies, all of which contribute to neurodegeneration and vascular injury linked to VaD [49]. In the MENA region, hypercholesterolemia is highly prevalent, with rates surpassing 30% in several countries, compared to the global average of 20–30% (**Figure 3**, Panel B). This disparity underscores the urgency of addressing LDL cholesterol management in the region to mitigate its role as both an independent and compounding driver of dementia risk.

### Environmental and genetic risk factors

#### Air pollution and climate

Emerging evidence implicates air pollution as a modifiable risk factor for dementia. Fine particulate matter (PM_2_._5_), nitrogen dioxide (NO_2_), and ozone (O_3_) have been associated with increased risk of cognitive decline and dementia in observational studies, primarily through mechanisms such as oxidative stress, neuroinflammation, cerebrovascular damage, and blood-brain barrier disruption [50]. The MENA region faces a unique combination of anthropogenic and environmental exposures, including industrial emissions, traffic-related pollution, and dust storms that contribute to elevated PM_2_._5_ concentrations in urban areas [51]. Climate change is expected to further exacerbate these exposures through more frequent extreme weather events, desertification, and worsening air quality [52]. One hypothesised mechanism involves inflammatory responses associated with high temperatures, which are more common amongst people living with dementia due to challenges in recognising heat risk and adapting to changing surrounding environments [52]. Although, most evidence linking air pollution to dementia risk derives from large observational cohorts conducted outside the MENA region, and direct region-specific longitudinal data remain limited, in many MENA capital cities the PM_2.5_ concentrations exceed WHO-recommended limits, underscoring the urgent need for regional air quality management strategies [53].

#### Genetic risk and consanguinity

Several genetic variants, most notably the APOE ε4 allele, have been associated with increased risk of Alzheimer disease in diverse populations [54]. However, evidence linking consanguinity to population-level risk of late-onset dementia remains limited, and most data relate to rare autosomal recessive neurodegenerative conditions rather than common late-life Alzheimer disease. Current evidence does not establish consanguinity as an independent population-level determinant of common late-onset Alzheimer disease, but rather suggests that it may increase the likelihood of homozygous expression of rare pathogenic variants in specific familial contexts.

That being said, the likelihood of AD or dementia can increase with having a genetic risk factor and the odds of disease development and chances of inheriting copies of such variants can be further augmented in cases of consanguinity. In the MENA region, high rates of consanguinity, estimated to be between 25–30% [55], may increase the probability of homozygous expression of rare variants associated with neurodegenerative disorders, although population-level effects on late-onset dementia remain incompletely characterised. Consanguinity increases the probability of homozygous expression of rare variants, several of which have been identified in familial dementia cases in MENA populations. Although consanguinity rates are declining in some MENA countries, the practice remains common - particularly in rural and socioeconomically disadvantaged communities - where cultural practices and family structures often drive these patterns [55].

## DISCUSSION

Dementia is rapidly emerging as a major public health challenge in the MENA region, not only because of demographic aging but because of a distinctive convergence of structural risk factors. While absolute prevalence remains lower than in many high-income countries (HICs), projections of up to a 367% increase by 2050 highlight the urgency of strategic risk management [3]. What differentiates MENA is not simply the pace of population aging, but the simultaneous layering of high cardiometabolic exposure, uneven educational attainment, persistent tobacco use, environmental stressors, and health system fragmentation. Together, these interacting exposures form a regional risk architecture, although the relative contribution of each determinant varies across subregions and income groups.

It is important to emphasise that the MENA region is highly heterogeneous [18,56]. Gulf Cooperation Council (GCC) countries differ markedly from Maghreb nations, Levant states, and conflict-affected settings in terms of income levels, health system capacity, demographic structure, and epidemiologic profiles. While regional patterns provide a useful macro-level framing, dementia risk distribution and care infrastructure vary substantially across countries. Interpretations throughout this review should therefore be understood as identifying broad structural tendencies rather than uniform conditions across all settings.

The heterogeneity of the region, reflected in World Bank income classifications (Figure S2, Panel A in the **Online Supplementary Document**), underscores the unequal distribution of economic and health burdens. High-income Gulf countries possess greater fiscal capacity to expand health infrastructure, but they remain constrained by high prevalence of metabolic risk factors [57,58], dependence on expatriate health workers [59], and persistent stigma surrounding dementia [3]. By contrast, lower-income countries face fragile health systems, low literacy, and limited diagnostic capacity, leaving many dementia cases unrecognised [10]. Despite these economic differences, certain challenges remain universal: stigma, low public awareness, and institutional unpreparedness are common threads across the region [5].

The economic burden of dementia further illustrates the divergence between global and regional patterns. In HICs, costs are dominated by direct expenditures on medical and social services [2]. In MENA, by contrast, expenditures are overwhelmingly concentrated in informal caregiving, as illustrated in our consolidated cost comparison (Figure S2, Panels B and C in the **Online Supplementary Document**). This imbalance reflects the near absence of formal long-term care infrastructure [4], leaving households to absorb the bulk of costs. Such invisible expenditures are rarely captured in national accounts, yet they represent a profound social and economic burden. The implications for gender equity warrant further consideration: women disproportionately shoulder caregiving responsibilities, often with limited institutional or financial support [5]. Thus, the dementia burden in MENA is not merely biomedical but also deeply social, embedding itself in family structures and reinforcing cycles of inequity.

Beyond cost structures, modifiable risk factors play an especially prominent role in shaping dementia trajectories in MENA. Taken together, the dementia risk landscape reflects the convergence of behavioural, social, cardiometabolic, environmental, and genetic exposures that interact to magnify vulnerability well beyond demographic aging alone. Lifestyle transitions marked by persistent tobacco use, widespread physical inactivity, and Western-Style dietary patterns have created a synergistic environment of vascular, metabolic, and neuroinflammatory stress [21,28,60]. These processes are compounded by social isolation, untreated hearing loss, and persistent educational disparities, all of which reduce cognitive reserve and heighten susceptibility to decline [1,61]. Cardiometabolic risks - hypertension, diabetes, obesity, and hypercholesterolemia – rarely occur independently; rather, they cluster to form a reinforcing triad of vascular and metabolic dysfunction, explaining much of the region’s projected surge in dementia burden [62]. Environmental exposures, including air pollution, recurrent dust storms, and extreme heat, further intensify these mechanisms [52], while high consanguinity rates amplify inheritance of rare genetic variants [55]. Unlike HICs, where age-specific dementia incidence has stabilised through improved education and risk factor management, MENA mirrors other low- and middle-income regions in facing a dual challenge: rapid population aging coupled with a disproportionately high prevalence of modifiable risks. This confluence positions MENA not only as an epicentre of future dementia growth but also as a critical case study of how layered biological, social, and structural determinants interact to shape cognitive aging.

The contribution of this review lies in synthesising these factors within a unified regional framework rather than treating them as isolated exposures. Much of the global literature evaluates single risk factors independently; however, in MENA, cardiometabolic clustering (obesity-diabetes-hypertension) [63], gendered educational disparities, informal caregiving dominance, and environmental pressures operate simultaneously and interactively. This clustering of modifiable exposures has been highlighted in global dementia prevention frameworks [1,64]. By examining these intersecting determinants across epidemiologic, economic, and health system domains, this review highlights how dementia vulnerability in MENA is structurally embedded within broader social and metabolic transitions. This integrative perspective may inform prioritisation strategies tailored to subregional contexts such as Gulf Cooperation Council countries, Maghreb nations, and conflict-affected settings.

The findings of this synthesis have implications for public health policy, however, such differs across subregional contexts. In high-income GCC countries, integrating dementia prevention into existing cardiometabolic screening programmes may be feasible given strong primary care infrastructure. In contrast, lower-income and conflict-affected settings may require foundational investments in surveillance, workforce training, and community-based awareness before specialised dementia services can be scaled [56]. Dementia prevention in MENA cannot be approached as a stand-alone programme but must be embedded within existing NCD frameworks. Hypertension, diabetes, and obesity are already major priorities across the region, and integrating dementia risk reduction into these programmes would be both cost-effective and scalable [1]. Strengthening primary care may be a key strategy for integrating dementia risk reduction: most countries remain ill-equipped to provide routine screening, management of vascular risk factors, or early dementia diagnosis [65]. Public awareness campaigns may play an important role in addressing stigma and fatalistic beliefs, while policy can address structural inequities such as gender gaps in education and access to health services, both of which are closely tied to dementia vulnerability. Based on the evidence synthesised above, several potential policy considerations emerge.

Prioritisation of dementia prevention strategies in MENA should reflect subregional heterogeneity. In high-income Gulf Cooperation Council (GCC) countries, where primary care infrastructure and cardiometabolic screening programmes are relatively well-developed, integrating dementia risk reduction into existing hypertension, diabetes, and obesity management platforms may represent a feasible near-term strategy [1,58]. In lower-income Maghreb and Levant countries, strengthening surveillance systems, workforce training, and community-based awareness programmes may be more immediate priorities before specialised dementia services can be scaled [10,13]. In conflict-affected and fragile settings, foundational investments in health system stability, data infrastructure, and culturally adapted primary care models are likely prerequisites to effective dementia prevention programming [66]. Across all contexts, strategies must account for gender inequities, informal caregiving structures, and resource constraints to avoid importing high-income policy templates that may not align with local care systems [1,5].

### Limitations

Critical research gaps contribute to constraints in progress. Few longitudinal cohort studies exist in the region, limiting knowledge of incidence, progression, and causal pathways. Furthermore, because this review relied on English-language peer-reviewed publications and available epidemiologic data, countries with limited research infrastructure or non-indexed local reporting may be underrepresented, potentially introducing selection and publication bias [11]. Genomic studies underrepresent MENA populations, hindering biomarker discovery and precision medicine [67]. Despite the region’s unique vulnerability to desertification and extreme heat, little research has examined the intersection of climate change and dementia risk [68]. Addressing these gaps will require coordinated investment in regional surveillance systems and dementia registries linked to broader NCD monitoring frameworks [10].

Addressing dementia risk management challenges require multisectoral public health strategies. Establishing a regional dementia consortium could harmonise methods, foster multi-country collaboration, and facilitate culturally adapted interventions [69]. Simultaneously, capacity building in workforce training, digital health infrastructure, and service delivery is essential for translating prevention frameworks into practice. Integrated, equity-focused strategies may help MENA countries mitigate the projected increase in dementia burden and build resilient, culturally competent systems of care.

## CONCLUSIONS

Dementia is poised to become a detrimental health and societal challenge in the MENA region, with prevalence expected to rise sharply as populations age and modifiable risk factors remain highly prevalent. The evidence reviewed here highlights the region’s dual burden of rapid demographic transition and limited health system preparedness and infrastructure, compounded by sociocultural barriers that delay diagnosis and care. Meeting this challenge will require embedding dementia prevention and care into broader NCD strategies, with sustained investment in surveillance systems, workforce training, and more research investment. Equally important will be policies that address the upstream drivers of risk, including low educational attainment, physical inactivity, and cardiometabolic disease. By prioritising investment in early intervention and strengthening both formal care structures and community supports, MENA countries have an opportunity to mitigate the future impact of dementia and build more equitable and resilient health systems.

**Data availability:** All data used in this study are included in the article and in the **Online Supplementary Document**. No additional data are available.

## Additional material


Online Supplementary Document
