# Supplementary material for: Dementia in the Middle East and North Africa: an integrative narrative review of epidemiology, risk structure, and health system implications
Source: J Glob Health. 2026 Jul 17;16:04181. doi: 10.7189/jogh.16.04181 (PMC13377586; doi:10.7189/jogh.16.04181)
Supplement: Online Supplementary Document [file jogh-16-04181-s001.pdf]

Supplement to: Dajani I, Mahboob A, Ibrahim SS, Ibrahim RS, Sedighi M, Szirzadi, Mahinrad S, Sabatini S, Khatoonabadi AR, Prina M, Castro CB, Mantegna JL, Akkari PA, Hosseini AA, Martins RN, Burley CV, Dunne J, Stephan BCM, Sohrab HR, Chaari A. Dementia in the Middle East and North Africa: an integrative narrative review of epidemiology, risk structure, and health system implicationsJ Glob Health. 2026;16:04181.

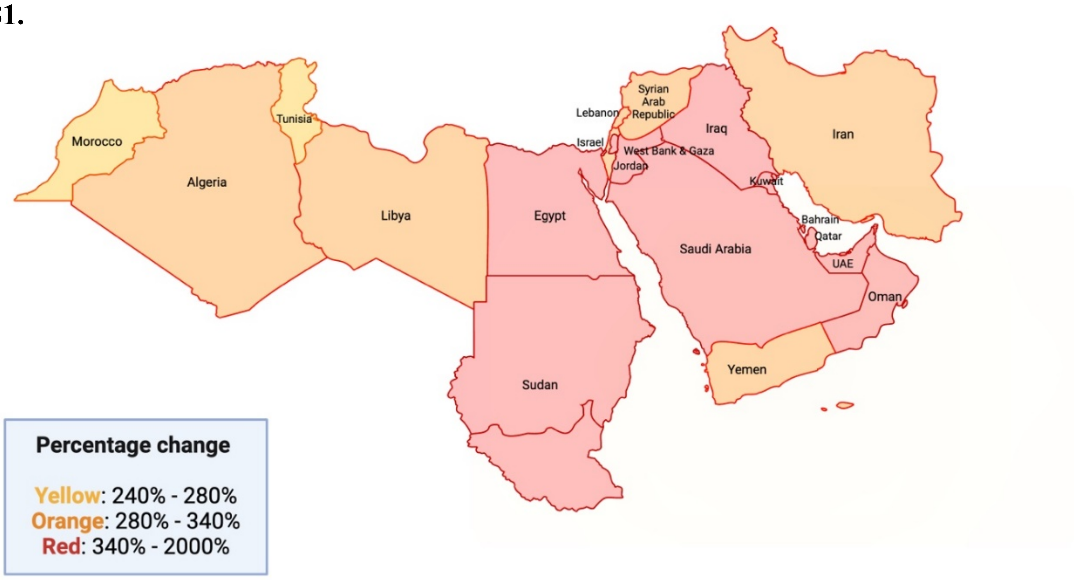

Figure S1. Projected percentage change in dementia prevalence by 2050 across the MENA region. Projections are based on Global Burden of Disease modelling assumptions and reflect demographic and epidemiologic trend extrapolations.

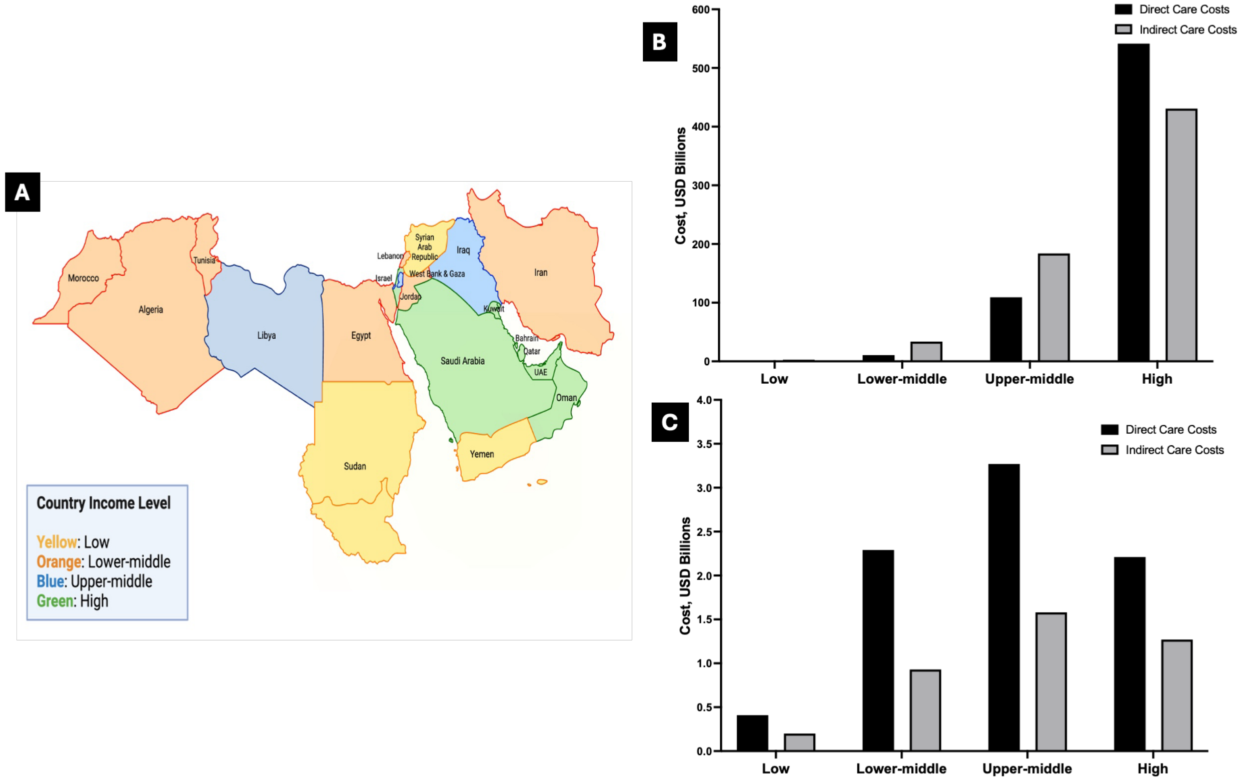

Figure S2.

- **A)** World Bank income classification of MENA countries (2021). The region spans low- to high-income settings, highlighting substantial economic heterogeneity. Despite this diversity, dementia prevalence remains high across the region, with many cases underdiagnosed due to weak health systems, limited public awareness, and stigma.
- **B)** Global dementia care costs in 2019, stratified by World Bank income groups. Costs are divided into direct (medical and social sector) and indirect (informal care) categories. In high-income countries, formal medical and social expenditures dominate, reflecting institutionalized care systems.
- **C)** Dementia care costs in the MENA region in 2021, stratified by income groups. Unlike global patterns, costs are predominantly driven by informal caregiving across all income categories, underscoring the absence of robust social sector services and the heavy reliance on family care networks.
